# Model Properties and Clinical Application in the Finite Element Analysis of Knee Joint: A Review

**DOI:** 10.1111/os.13980

**Published:** 2024-01-04

**Authors:** Mingyue Yan, Ting Liang, Haibo Zhao, Yanchi Bi, Tianrui Wang, Tengbo Yu, Yingze Zhang

**Affiliations:** ^1^ Department of Orthopedics The Affiliated Hospital of Qingdao University Qingdao China; ^2^ Institute of Sports Medicine and Health Qingdao University Qingdao China; ^3^ Department of Orthopedic Surgery Qingdao Hospital, University of Health and Rehabilitation Sciences (Qingdao Municipal Hospital) Qingdao China; ^4^ Department of Orthopedics The Third Hospital of Hebei Medical University Shijiazhuang China

**Keywords:** biomechanics, clinical application, finite element model, knee joint, material properties

## Abstract

The knee is the most complex joint in the human body, including bony structures like the femur, tibia, fibula, and patella, and soft tissues like menisci, ligaments, muscles, and tendons. Complex anatomical structures of the knee joint make it difficult to conduct precise biomechanical research and explore the mechanism of movement and injury. The finite element model (FEM), as an important engineering analysis technique, has been widely used in many fields of bioengineering research. The FEM has advantages in the biomechanical analysis of objects with complex structures. Researchers can use this technology to construct a human knee joint model and perform biomechanical analysis on it. At the same time, finite element analysis can effectively evaluate variables such as stress, strain, displacement, and rotation, helping to predict injury mechanisms and optimize surgical techniques, which make up for the shortcomings of traditional biomechanics experimental research. However, few papers introduce what material properties should be selected for each anatomic structure of knee FEM to meet different research purposes. Based on previous finite element studies of the knee joint, this paper summarizes various modeling strategies and applications, serving as a reference for constructing knee joint models and research design.

## Graphical abstract

This review summarizes various modeling strategies and clinical research on the knee joint to serve as a reference for constructing knee joint models and designing research.

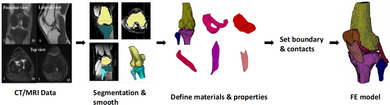

## Introduction

The knee joint is the stress‐bearing joint with the most complex structure and the highest functional requirements among all the joints in the human body. The knee joint is supported by the femur, tibia, fibula, and patella and is surrounded by soft tissues such as the joint capsule, meniscus, ligaments, tendons, and muscles. There are many ligaments in the knee joint to improve stability, including the medial collateral ligament (MCL), lateral collateral ligament (LCL), anterior cruciate ligament (ACL), posterior cruciate ligament (PCL), medial patellofemoral ligament (MPFL), lateral patellofemoral ligament (LPFL), and patellar ligament (PL). Because of long load‐bearing times and a large amount of exercise, traumatic and non‐traumatic factors can cause damage or degeneration of the knee joint, resulting in meniscus injury, ligament rupture, or osteoarthritis.

To better understand the knee joint's function and disease mechanism, many biomechanical studies have been carried out based on cadaveric specimens. The advantage of cadaveric research is that it is more intuitive; however, it is not helpful for in‐depth research.^1^, ^2^, ^3^ First, it is difficult and expensive to obtain cadaveric specimens. Second, large joints are challenging to preserve, significantly limiting intact joint research. Third, complex joint movements are difficult to simulate. With the advancement of digital medicine and computing software, FEA can better solve the pain points of cadaver research and living body research in biomechanics analysis. Researchers can study the mechanical response of tissues in a non‐invasive way, simulating human mechanical tests that cannot be performed at relatively low cost and risk. In addition, researchers can conduct repeated research on the constructed finite element model (FEM) and make the research results more intuitive by visually analyzing the internal structure of the joint, which can be used to study the mechanical changes of prothesis and surgical implants. Many researchers have successfully simulated the main structure of the knee joint through FEA and established a variety of FEMs,^4^, ^5^ achieving worthwhile and meaningful results.

However, few papers provide guidance on the selection of material properties for each anatomical structure of knee FEMs to cater to diverse research objectives. Drawing from previous studies in finite element analysis of the knee joint, this paper presents a comprehensive overview of various modeling strategies and their applications, serving as a valuable reference for constructing knee joint models and designing research investigations.

## Materials and Methods

**FIGURE 1:**
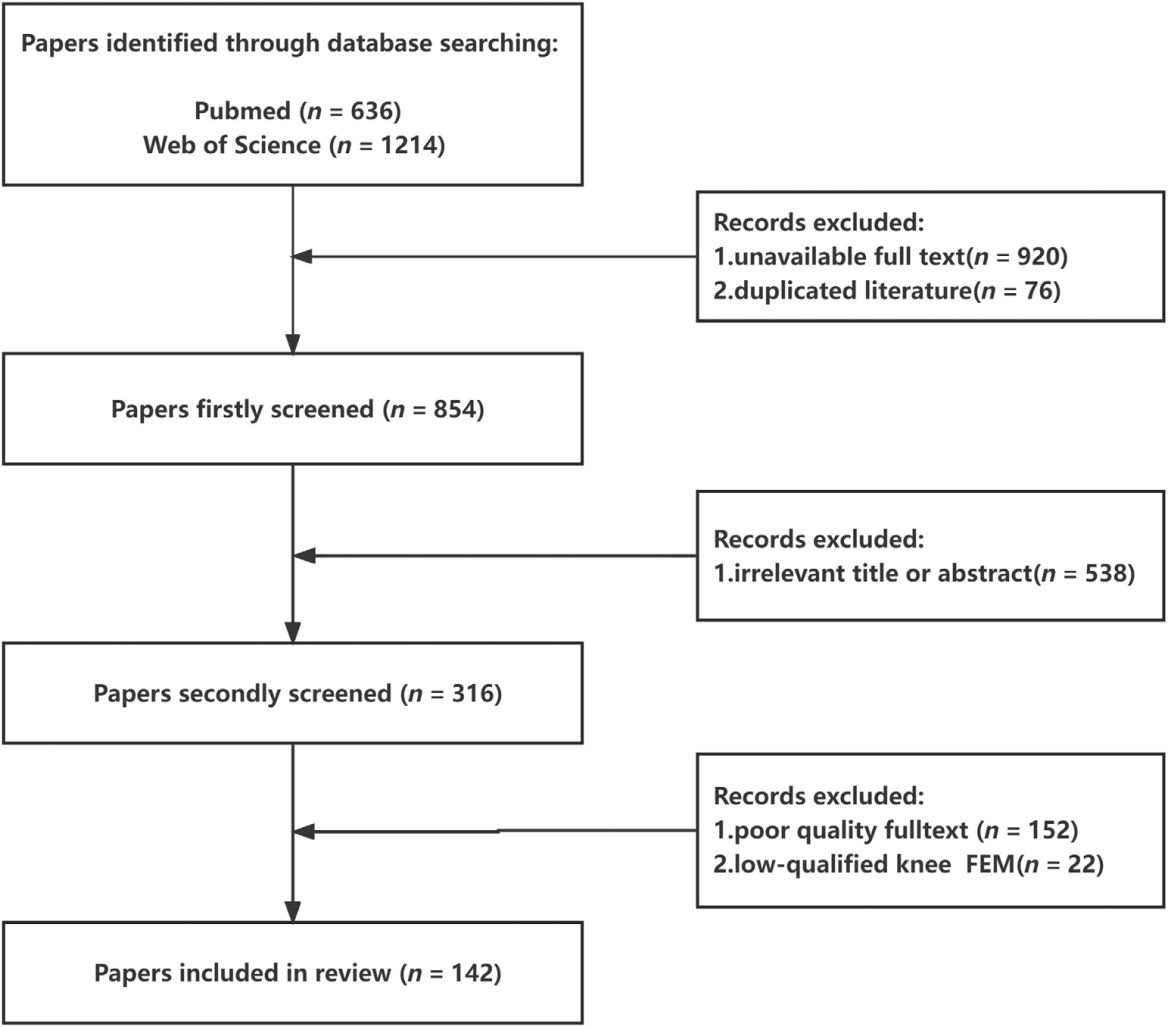
The flow chart of literature retrieval and screening methods.

Publications related to FE modeling and simulation of knee joints were identified using PubMed and WOS search engines. The keywords “knee joint” and “finite element model” were selected to search the literature of the last 10 years based on titles and abstracts. We only selected articles written in English. The papers were then filtered to select papers that met the following two basic criteria: (i) a three‐dimensional (3D) FEM of the knee joint is studied, or (ii) the topic of the study is the material properties of the anatomical structure that is used in the FEM. The exclusion criteria: (i) unavailable full text, (ii) irrelevant title or abstract, (iii) poor quality full text, and (iv) low‐qualified knee FEM. A total of 1850 articles were retrieved and 142 articles were selected according to the exclusion criteria (Figure 1). Based on previous FEMs of the knee joint, we discuss two main aspects of finite element research, including model construction and clinical application.

## Model Construction

### Modeling Tools

**FIGURE 2:**
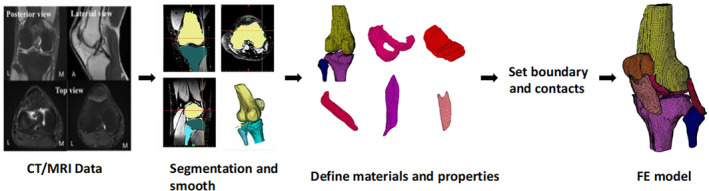
Overview of the modeling and simulation approach for creating FE models.

The workflow of finite element modeling is shown in Figure 2. The first step of FEM construction is to obtain high‐quality medical imaging data. X‐ray, ultrasound, CT, and MRI are widely used to detect bone, joint, and soft tissue diseases. Because the establishment of FEMs involves the 3D reconstruction of tissues, CT and MRI are the primary sources of image data for finite element modeling. The parameters for CT and MRI scanning are closely related to the fineness of the FEM. The slice thickness of CT and MRI is usually 1 mm. The thinnest CT layer thickness is 0.5 mm.^6^ Wang *et al*. (2020) used a 3.0T FLASH pulse sequence to perform MRI scanning, which made the slice thickness reach 0.2 mm.^7^

CT images can clearly show the morphology and lesions of bone tissue, but the soft tissue is poorly displayed. Based on CT images, researchers usually build FEMs of bony structures such as the femur, patella, tibia, and fibula.^8^ MRI images can clearly display cartilage and soft tissue morphology, which are used to construct FEMs of cartilage, meniscus, ligament, and muscle tendon.^9^ The bone tissue model acquired by MRI is not as refined as that obtained by CT scanning. Considering the radiation of CT, some researchers choose MRI to outline the morphology of bone when performing finite element modeling of bone tissue in children.^10^ John *et al*. tried to outline the medial collateral ligament from CT, but the CT images failed to clearly show the boundaries of the ligament and meniscus,^11^ which means MRI images are still the first choice for obtaining the delicate structures of soft tissues such as ligaments and menisci. The parameter settings of CT and MRI machines are different, and the accuracy of the model might be affected if the extracted 3D structures are directly joined together. Some researchers placed external reference points on the knee joint of volunteers to match CT and MRI images and then combined different structures of the knee joint by anatomy.^12^

**FIGURE 3:**
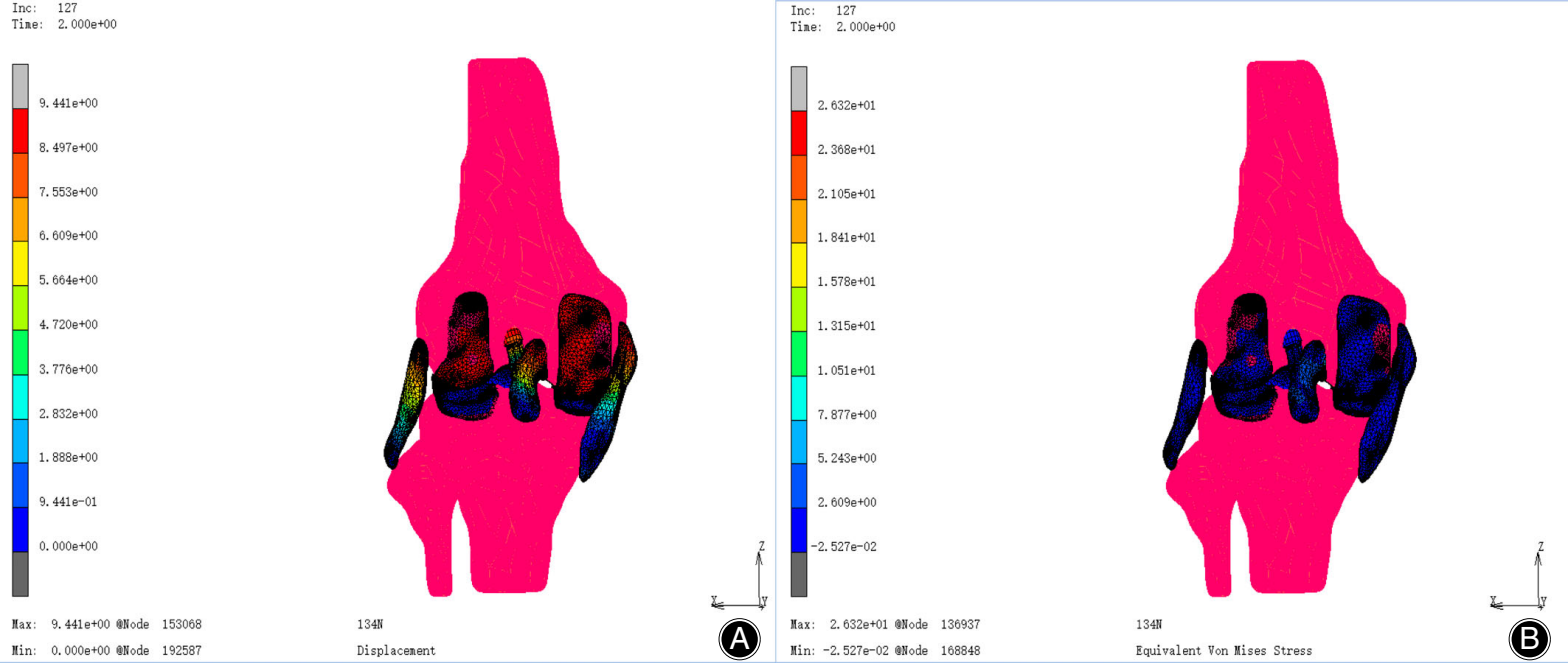
Finite element model (FEM) analysis results under load. (A) contour result of displacement of various anatomic structures under load. (B) Contour result of stress on various anatomic structures under load.

Most tissue models need to be manually outlined on the images by professionals using image processing software such as Mimics,^13^ then imported into finite element analysis software such as ABAQUS and MARC for calculation.^14^ In this analysis and calculation process, parameters such as displacement and stress can be obtained (Figure 3). Researchers developed Convolutional Neural Networks (CNNs) for MRI automatic segmentation by using semi‐supervised learning, thus significantly improving the efficiency of model construction.^15^ Similarly, Yin *et al*. proposed an automatic image segmentation method based on Python, and the obtained bone and cartilage were within a 0.2 mm error compared to manual outlining.^16^

In general, FEM outlining needs to be based on high‐quality images, relying on a high‐performance computer and related software for image processing and finite element analysis. Semi‐automatic or fully automatic segmentation of FEMs might be a potential development direction.

### Material Properties

In the musculoskeletal system of the knee joint, bone, cartilage, and soft tissues such as ligaments and meniscus are mainly responsible for supporting body loads, limiting joint movements, and protecting the joint. The human body is far more complex than a sophisticated machine, and the mechanical properties of soft tissue are probably more advanced than most engineered materials. Essentially, the tissues of the musculoskeletal system are composed of fibers, proteins, water, and electrolytes, which are ingeniously combined to give each tissue a specific mechanical function.^17^, ^18^ Proteoglycans, associated with osmotic and swelling effects, can affect the viscoelasticity and other characteristics of tissues after combining with water.^19^ Collagen fibers give the tissue a certain amount of tensile strength.^20^

Biomechanical experiments are required to obtain mechanical parameters for the FEM, and water loss can significantly affect tissue structure and mechanical properties. It is revealed that ligaments and tendons could be preserved at −20°C before biomechanical tests,^21^ providing a reference for tissue preservation and subsequent mechanics experiments. In addition, the joint's kinematics state would affect the choice of material properties. The static analysis of biomechanics applies to transient changes or equilibrium states, which can be used to study the characteristic changes of soft tissues at a certain point in time. Because time‐dependent behaviors such as creep require continuous stress for a certain period,^22^ appropriate simplification can be considered in static analysis.

In conclusion, appropriate material properties and material parameters can better describe the mechanical properties of soft tissues in the process of finite element modeling, which is also crucial to improve the accuracy of the FEM. The material properties in current finite element studies are mainly derived from other studies and material tests. The model materials for each structure of the knee will be described separately below.

#### Bone

Bone tissue is the most rigid connective tissue in the body, composed of cells, fibers, and large amounts of calcium salts. Because the density and Young's modulus of bone tissue are much greater than that of the surrounding soft tissue, most researchers model bone as a rigid body in finite element studies.^22^, ^23^, ^24^ A study has shown that rigid bone models have reliable accuracy for 3D bone model reconstruction.^25^ However, it has also been suggested that as long as the bone is modeled as a rigid body, cartilage stresses and strains would change significantly, especially in the medial compartment.^26^

To calculate the stress of tissues such as ligaments or grafts on bone tissue, it has been proposed that bone be modelled as a linear elastic material.^27^ The elastic modulus and Poisson's ratio of cortical bone and cancellous bone need to be defined separately to make the linear elastic bone tissue more realistic.^28^ The bone model of osteoporosis needs a relatively low Young's modulus.^29^ In addition, Schileo *et al*. proposed that the elastic modulus of bone tissue can be obtained from the density–elastic modulus relationship, which has been proved to predict the strain accurately.^34^ Researchers used dual‐energy CT to obtain bone density and then used the validated density–elasticity relationship to calculate a specific Young's modulus for each element.^30^ Gujar and Warhatkar (2020) introduced an image processing technique that can reduce a significant amount of modeling time based on bone density in CT images, thus deriving Young's modulus to investigate the biomechanical properties of the proximal femur bone.^31^

In general, the rigid model is sufficient as the constitutive relation of bone tissue in most cases, and it can reduce the calculation cost. The choice of linear elastic material for bone tissue studies involving complex loads would make the results more reliable. Researchers must choose an appropriate material model based on research conditions and purposes.

#### Cartilage

Articular cartilage contains large amounts of water, proteoglycan, and collagen fibers. The cartilage thickness on the joint surface is variable, and simplifying the cartilage to a model with the same thickness will cause a significant increase in cartilage contact stress.^32^ Commonly used cartilage models include monophasic, biphasic, and triphasic models. The monophasic model assumes that the articular cartilage has only one solid phase, which follows the elasticity theory. The biphasic model suggests that articular cartilage is composed of an incompressible solid phase and a liquid phase, consistent with the fact that cartilage contains a large amount of water and a porous structure.^22^ The triphasic model considers the fixed charge attached to the cartilage matrix and adds an ionic phase to the biphasic model.^33^ The monophasic elastic and biphasic porous models are used in the current study.

The monophasic elastic model of cartilage is common in finite element studies. Articular cartilage is often modeled as isotropic linear elastic materials, the simplest way to describe mechanical properties in soft tissue. This model can significantly reduce calculation costs in complex contacts, primarily when the research does not focus on the tissues near the contact. Beillas *et al*. demonstrated that variations in the material properties of cartilage within the normal range significantly affect the pressure at the cartilage contact surface.^34^ The higher the elastic modulus and Poisson's ratio of cartilage tissue, the higher the maximum pressure value of the contact surface.^34^ A method for calculating Young's modulus of cartilage from the T2 relaxation value revealed that the homogeneous material model might underestimate the tissue strain in the damaged area and overestimate the strain in the healthy area when predicting the occurrence of osteoarthritis (OA).^35^ Essentially, cartilage is also a viscoelastic structure, and the collagen fibers in articular cartilage have viscoelastic behavior.^36^ However, considering that the time of standing on one leg is very short compared with the viscoelastic time constant of cartilage, most studies did not consider the viscoelastic properties of articular cartilage, assuming that cartilage is an isotropic linear elastic material, which can make the calculation more efficient.

Healthy articular cartilage is water rich, and the fluid contents can undergo exudation and osmosis under load, suggesting that the cartilage structure is time dependent. Most researchers now believe that the biphasic model is the most appropriate model to describe the mechanical behavior of articular cartilage. Fibril‐reinforced poroviscoelastic (FPRVE) is a type of biphasic model. The collagen fibers in the solid phase are considered viscoelastic materials, and the non‐fibrous portion is hyperelastic material. The FRPVE model is probably the most advanced for cartilage materials and can effectively predict the mechanical behavior and degenerative response of fibrous and non‐fibrous matrices in articular cartilage.^37^

In general, the choice of cartilage model material is related to the research purpose. The monophasic model can effectively simulate global changes, which is recommended for the study of joint motion. The biphasic model is more accurate in quantifying local stresses, so fine cartilage models might be essential for conducting studies that simulate failure and injury, such as osteoarthritis.

#### Ligaments

Knee ligaments contain a large amount of water, elastin, and collagen fibers. The proteoglycan and hyaluronic acid in the matrix can absorb water from the surrounding area to provide particular support, and the ligaments need to be considered incompressible when modeling. There are significant individual differences in the material properties of ligaments. Gardiner *et al*. proposed that a ligament model with average material properties would not differ significantly from a specific model in predicting stress–strain.^13^ The most widely used ligament models in current research are the incompressible spring model and the hyperelastic model.

Considering that the stress–strain curve of the knee ligament is not linear,^38^ many researchers have modeled ligaments as transversely isotropic and incompressible hyperelastic materials with material parameters consistent with the strain energy density function of large strain theory.^39^, ^40^ In addition, it has been proposed that ligaments are viscoelastic tissues, and the Kelvin–Voigt model is a widely used viscoelastic model for ligaments and tendons.^41^ However, viscoelasticity can be ignored when the strain rate is very fast or slow in practical studies.^42^ At high strain rates where the ligaments do not have enough time to relax significantly, the viscoelastic model is approximate to the elastic model; at low strain rates where the ligaments are almost completely relaxed, the viscoelastic model is still essentially elastic.^42^ Therefore, it might not be necessary to account for its viscoelastic component when modeling ligaments.

In addition to treating ligaments as a hyperelastic material, they can also be regarded as a spring element and correlate the stress with the displacement.^27^ In a finite element study of the ligaments, the cruciate ligaments were modeled with two and collateral ligaments with three bundles of spring.^43^ It has been suggested that increasing the number of springs in the finite element study of high knee flexion could improve the accuracy of the analysis results of the cruciate ligament model, which better simulated the deformation and stretching behavior of the knee ligament and achieved a better dynamic assessment of the joint motion.^44^ The spring model simplifies the hyperelastic model, which can better predict joint kinematics while having calculation advantages.^45^ However, the spring model has certain drawbacks, it is difficult to simulate the state of ligament winding and curling, and it is impossible to study the biomechanics of the ligament itself.

Accurate model parameters depend on rigorous biomechanical testing. Peters *et al*. concluded that age needed to be considered when performing biomechanical experiments on ligaments, and the failure load of ligaments would decrease with the age and progression of OA.^46^ The total strain of an individual ligament is not the same as that of a bone–ligament–bone complex. The mechanical parameters obtained by treating the bone–ligament–bone complex as a functional unit are more suitable for FEMs.^47^ In addition, the pre‐stress of ligaments was neglected in many studies. It has been proposed that pre‐strain and pre‐stress in the ligament model could significantly alter the ligament's local load and the joint's stability, affecting joint kinematics and contact outcomes.^48^, ^49^

In general, the type of ligament model is related to the purpose of the study. The spring model has the advantage of simple modeling and low calculation, which could accurately predict joint kinematic performance. The hyperelastic model is more applicable to the biomechanical study of the ligament itself, and the interaction of the ligament with the surrounding tissues can yield a more accurate stress–strain.

#### Meniscus

The meniscus is a crescent‐shaped fibrocartilage located between the tibia and the femur, consisting of water, collagen fibers, and matrix. This allows for a more even distribution of pressure in the joint.^32^ The microstructure of the meniscus is anisotropic.^50^ Most studies suggest that the fibers of the meniscus are arranged in a circular or radial pattern.^51^ The material properties of the menisci are related to age and joint degeneration. Joint degeneration has little effect on the tensile properties of the meniscus but significantly affects the compressive properties, with a significant increase in axial stress–strain.^52^ The current FEMs of the meniscus are mainly linear elastic, hyperelastic, and fiber‐reinforced poroelastic (FRPE) materials, and there are few studies considering the viscoelasticity of collagen.

Mechanical properties of the meniscus are depth‐dependent.^53^ The properties and functions of cartilage and meniscus are somewhat similar, and some finite element studies have modeled the meniscus with a fiber‐reinforced porous elastic material. However, a study proved that the depth dependence of the meniscus had little effect on the mechanics of the cartilage.^54^ Considering the complexity of the FEM of the knee joint, the meniscus model should be appropriately simplified.

The isotropic linear elastic material is a simple material model for the meniscus. Because of the high stiffness of the meniscus in the fiber direction, calculating the strain in the fiber direction would produce a significant error, and some researchers considered the meniscus a transverse isotropic linear elastic material,^24^ which was similar to the histological fingdings.^55^ A study suggested that the meniscus showed heterogeneity at high load.^56^ Hyperelastic materials might make mechanics results more plausible.

In general, researchers can make certain simplifications to the meniscal model depending on the purpose of the study. The cartilage's thickness and the meniscus's shape and volume could significantly affect the accuracy of the FEM for the knee joint.^57^ Therefore, geometry is more important than material when modeling the meniscus, and transversely isotropic hyperelastic materials might be a better choice.

#### Muscle and Tendon

The muscle is composed of the muscle belly and tendon. The muscle belly is the main structure of the muscle, mainly consisting of skeletal muscle fibers and connective tissue; tendons are composed of dense connective tissue, located at the ends of the muscle belly to anchor the muscle to the bone. Accurate muscle morphology can be obtained with the help of magnetic resonance MR images.

Esrafilian *et al*. simplified the muscle model and used spring elements to simulate the muscle.^58^ Some scholars have also modeled the muscle as a two‐dimensional fiber‐reinforced structure containing membrane and spring elements.^59^, ^60^ Tendons are similar in structure and function to ligaments. Tendons can be modeled with spring elements and hyperelastic material models, with the spring elements being widely used.^58^, ^61^ Wang *et al*. compared three tendon models: Marlow, Neo‐Hookean, and linearly elastic models. They found that the Marlow model was more consistent with the experimental mechanical data of the patellar tendon.^62^

OpenSim software can assist researchers in building a set of musculoskeletal models. Models constructed by OpenSim could simulate musculoskeletal dynamics and neuromuscular control to study joint activities, calculate muscle forces, and predict joint movements without the need for experiments,^63^ but it is difficult to study the contact mechanics of joints. The FEM is an excellent complement to the musculoskeletal model, and the combination of the two models can be used to analyze the dynamic biomechanical performance of the knee joint.^64^ OpenSim's optimization toolbox can be used to evaluate muscle forces to generate specific FRPE models.^65^ The musculoskeletal model could be established with the help of electromyography (EMG). Researchers compared the reliability of different musculoskeletal models to predict joint motion, and the EMG‐assisted musculoskeletal model was able to better assess the kinematics and stress load distribution in the knee joint.^48^, ^66^ A rapid musculoskeletal‐finite element (MS‐FE) modeling method based on EMG has been introduced and validated. EMG‐assisted musculoskeletal models enable more accurate predictions and can be applied to knee joint dynamics studies.^67^

Overall, musculoskeletal models provide non‐invasive insight into the interactions between muscle mechanics and knee kinematics, which cannot be measured in the laboratory. The musculoskeletal model combined with the FEM provides the basis for solving complex biomechanical problems and lifts the finite element study of the knee joint to a new level.

### Contact Conditions

The FEM involves contact between multiple structures. Combining the corresponding structures according to the anatomical relationship could make the model analysis results more reliable. The attachment of the meniscus root to the tibia could be simulated by a spring.^68^ The ligament–bone connection can be made by specifying the last row of the ligament's mesh as the same material as the bone tissue.^11^ The meniscus could be connected to the ligament using a linear non‐compression spring element.^69^

Contact friction is a challenging problem in finite element studies of the knee joint. The coefficient of friction within the joint is low, and most FEMs of the knee assumed no friction within the joint. There is no consensus on the coefficient of friction between cartilage.^70^, ^71^, ^72^ It has been suggested that the coefficient of friction of the knee joint with lubrication of the joint fluid and hydration of the cartilage is approximately 0.01,^73^ and 0.02 was also used in some studies.^74^ Finite element software provides a variety of algorithms for the simulation of frictionless and frictional contact.^75^ However, contact calculations between soft tissues might not converge well, especially when the soft tissue contact surface undergoes large deformation. Ateshian *et al*. suggested that the FEBio software might be able to solve this problem.^76^

### Model Verification

Model validation is used to verify the calculation results of the simulation model, which can assess the credibility of the FEM. The data for the model validation was mainly obtained from biomechanical experiments or references,^24^, ^77^ but the verification of purposeful experiments would make the model more credible.^78^

The design of verification experiments is mainly based on the research purpose and the variables concerned. Many different experimental methods can be applied in the study to obtain verification data. The dynamic fluoroscopy device can obtain the position of the bony structure in motion.^59^ *In vitro* biomechanical devices can attain biomechanical data such as kinematics by recording the body's load on the ground and the trajectory of the movement.^59^ Knee joint kinematics can be evaluated with specific motion capture systems and mechanical acquisition devices to obtain kinematic and mechanical data.^79^ The model can be verified by comparing the displacement, rotation, contact area, and contact pressure with the knee joint.^80^ Local variables such as stress–strain can be obtained by implanting pressure sensors in ligaments.^81^ Knee prosthesis with sensors also provide data such as joint surface stress.^82^ EMG signals enable the activation of muscles and serve as an objective evaluation indicator for muscle models.^77^ Comparing the experimental data with the prediction results of the FEM by linear regression is efficient in evaluating the model's accuracy.^83^

## Application and Characteristics

Finite element models help us better understand diseases’ pathogenesis and provide efficient support for clinical application and rehabilitation exercises.

### Clinical Application

The application of FEMs is described below separately using the classification of knee joint diseases.

#### Bone Defects and Fractures

Bone is the main load‐bearing structure of the knee joint, and FEMs containing bone tissue help understand the biomechanical function of the knee joint and the mechanisms of disease.

Finite element analysis is widely used in the study of bone and cartilage defects.^84^ Using cancellous bone grafting and large bone grafting for bone defects under the tibial prosthesis might cause greater micro‐movement and higher stress on the prosthesis, which could be improved by using a stiffer graft material, such as compressed cancellous bone.^85^ Liu *et al*. compared the results of metal blocks and cement screws for tibial defects and found that cement screws were more compatible and suitable for minor bone defects; metal blocks were stronger and ideal for large bone defects,^86^ with better results for personalized and customized metal blocks.^87^ Bone defects in total knee arthroplasty (TKA) revisions might be treated with a prosthesis with conical mesh, which could reduce the stress load between the prosthesis and the bone cement.^88^ Using finite element analysis, Zhao *et al*. evaluated the biomechanical differences in high tibial osteotomy (HTO) using reverse screws and bone grafting.^89^ Both reduced the stress on the tibia, but there was a risk of plate fracture with reverse screws.^89^ Implantation of osteochondral grafts might be helpful in cartilage defects. Kilicaslan *et al*. performed a finite element study on cartilage grafts, and the mismatch between the cartilage thickness of the graft and the cartilage surface had little effect on the biomechanics of the joint when the graft was flush with the cartilage surface.^90^

Finite element analysis could also be used in bone fracture research and treatments. MacLeod *et al*. developed a new HTO plate, a large span plate that significantly reduced the range of high skeletal strain and increased the movement between fracture fragments.^91^ Ren *et al*. proposed a plate for the treatment of posterolateral tibial plateau fracture (PLTPF) through an anterolateral approach and compared the new plate with a conventional one, verifying that the new plate was more convenient to operate and had better biomechanical properties.^92^ Du *et al*. compared the clinical effects and mechanical properties of different treatment methods for inferior pole fractures of the patella.^93^ The anchor‐loop plate had better biomechanical stability and clinical effect than the Kirschner wire tension band combined with patellar cerclage.^93^

Overall, finite element analysis of the knee joint applied to fractures and bone defects can simulate the biomechanical results well and has good prospects for the research and treatment of fractures and bone defects.

#### Osteoarthritis

The cartilage of the knee joint might change its structure, and mechanical behavior under repetitive high loading and FEMs can help us understand cartilage damage and degeneration in a more in‐depth manner. The integrity of the cartilage base affects the stability and load‐bearing capacity of the joint, and damage to the cartilage base might occur first in superficial layers during joint degeneration.^94^ Anwar *et al*. analyzed the effect of subchondral bone cysts on knee joint biomechanics. Larger bone cysts could increase the local stress on the articular cartilage, and multiple cysts or excess weight would aggravate knee degeneration.^95^ Fadi *et al*. developed a musculoskeletal model to study lower limb muscle strength and knee joint stress in average‐weight and obese people, finding that obese people had significantly greater muscle strength, greater relative motion of the medial compartment in the knee, and relatively more significant cartilage stress.^96^

Arjmand *et al*. proposed an FEM to accurately quantify the changes in mechanical parameters in normal populations and patients with OA,^97^ finding that the bone stress in the proximal tibia was higher in patients with OA, which might be necessary for understanding the pathogenesis of knee OA.^97^ Peter *et al*. explored the influence of the morphology of the distal femoral epiphysis surface on the epiphysis biomechanics through finite element modeling and found that a complex‐shaped articular surface could reduce the stress on the epiphysis during knee flexion.^98^

Surgical treatment of knee osteoarthritis often includes arthroplasty or osteotomy. FEMs can be used to guide clinical decision‐making by evaluating cartilage. An FEM has been designed to accurately assess the need for revision by analyzing the clinical characteristics and cartilage stress of unicondylar knee arthroplasty (UKA) patients.^99^ Changes in knee joint force lines can also affect the cartilage. Nakayama suggested that severe knee valgus was unsuitable for HTO, as the tibial cartilage would generate excessive shear stress and thus affect the effectiveness of the surgery.^100^ Further, finite element analysis can assist in designing and selecting knee prosthesis, guiding surgeons to optimize the operation in time. Finite element studies of knee protheses suggest that musculoskeletal models would affect the knee joint's kinematic and kinetic outcomes.^101^ Shu *et al*. proposed an FEM verified by *in vivo* fluoroscopy, pressure sensors, and EMG for preoperative testing of a newly invented knee prosthesis.^59^ Fernando *et al*. suggested that reducing the tibial prosthesis size could prevent excessive prosthesis internal rotation.^102^ Koh *et al*. compared the kinematics of different tibial protheses for UKA, and the femoral kinematics performed better with the convex surface prosthesis.^103^ All‐polyethylene knee protheses have good biomechanical characteristics in a relatively young population, and surgeons can consider relaxing the age limit.^104^ Medial pivot knee prostheses have less medial stress and better kinematic performance, but less femoral internal rotation might cause pain in the anterolateral aspect of the knee.^105^ A finite element analysis of the knee femoral prosthesis stems found that cemented fixation had an advantage over press‐fit fixation in terms of knee stability and stress shielding and that the short stem prosthesis had the lowest stress‐shielding effect.^29^ A biomechanical study was conducted on whether PCL was preserved in TKA, finding that the stresses on the femoral and tibial prothesis increased with the femoral flexion angle and that the greater the PCL stiffness, the greater the stresses on the tibial prosthesis and ligaments.^106^

Finite element analysis can also be a tool for the study of the biomechanical effects of the implant angle of the prosthesis on the knee joint.^107^ Innocenti *et al*. found that adjusting the alignment of the tibial prosthesis resulted in a more significant variation in loading, suggesting that it would be safer for the surgeon to adjust the alignment of the femoral prosthesis.^108^ The influence of UKA tibial prosthesis alignment and coronal plane inclination on the biomechanics of the lateral compartment has been analyzed. The tibiofemoral cartilage was subjected to the most significant contact stress and load proportion when the prosthesis was valgus, suggesting that a slight varus or neutral position of the prosthesis could delay the development of lateral compartment osteoarthritis.^109^ However, excessive varus of the prosthesis could increase the lateral compartment load and accelerate the progression of osteoarthritis.^110^ Danese *et al*. suggested that the UKA tibial prosthetic varus alignment could reduce cancellous bone tension but increase the stress on the anteromedial cortical bone.^111^

The FEM can be used to evaluate the outcome of knee replacement. Kang *et al*. explored the effect of posterior tibial slope (PTS) on biomechanics after TKA.^112^ Increased PTS resulted in increased joint mobility, but a greater PTS would decrease quadriceps muscle strength and collateral ligament tension and result in tibiofemoral joint loosening.^112^ Kwon *et al*. suggested that adjusting the PTS could regulate the anterior–posterior displacement of the tibiofemoral joint after UKA, implying that patients deficient in ACL or PCL could also undergo UKA.^113^ The posterior condylar offset (PCO) increases the stress on the quadriceps, patella, and patellar tendon when moving anteriorly, but increasing the PTS would reduce the stress and thus offset the negative effect of the PCO moving forward.^114^ The patient‐specific technique of joint replacement is to improve the coverage of the prosthesis by removing the least amount of bone according to the patient's condition. Kang *et al*. concluded that patient‐specific UKA would affect the kinematic performance of the knee but could reduce contact stress in the tibia and meniscus,^115^, ^116^ effectively slowing the progression of OA. In addition, femoral fractures might occur in the drilled holes left over from navigational TKA surgery. Sun *et al*. combined the results of finite element analysis with biomechanical experiments to propose a safety zone for drilling.^117^ Shinji *et al*. studied the risk of medial tibial condyle fracture in UKA, which increased with tibial valgus and posterior tibial sagittal incision enlargement.^118^ Stoddart *et al*. suggested that bi‐UKA had a similar risk of tibial eminence avulsion fracture to UKA, with a higher risk of fracture when tibial bone density was lower.^119^

To conclude, finite element analysis applied to osteoarthritis and joint replacement can provide strong biomechanical evidence for prosthesis design and placement, as well as predicting the outcome of replacement surgery. Therefore, it has a promising future in clinical research.

#### Meniscus Injury

The meniscus has a high probability of injury during sports, and finite element studies mainly focus on the injury and repair of the meniscus. The constitutive model of the meniscus can be established using the finite element analysis software.^120^ The medial meniscus was found to have the stiffest anterior horn and the most significant nonlinear features.^121^ The meniscus compression degree and stress distribution could be analyzed with the help of FEMs. Simulating the displacement and deformation of the meniscus during motion could provide a better understanding of the mechanism of meniscus injury.^122^ It has been proposed that stress would shift to the affected side after meniscal tears and that stress and shear forces in the meniscus and cartilage would increase after lateral meniscal tears, with medial injuries showing more significant changes.^12^, ^123^ Wang *et al*. found that a radially wider incomplete tear of the medial meniscus could disrupt the circumferential stress transmission in the meniscus and that higher stresses at the end put the meniscus at risk of complete rupture.^124^ It has been suggested that the maximum contact stress on the articular cartilage after meniscectomy was approximately twice that of a healthy joint,^125^ with a significant increase in stress–strain on the lateral cartilage,^126^ which might help to explain the cartilage damage and degeneration observed after meniscectomy. Meniscus replacement might delay long‐term adverse events such as OA.

Finite element models can also evaluate the effectiveness of meniscal repair or grafting. Wang *et al*. compared different methods of repairing posterior root tears of the lateral meniscus, and the double‐needle technique allowed for better clinical results.^80^ Kim *et al*. compared the surgical results of meniscal transplantation *via* the parapatellar approach and the transpatellar approach, with the latter demonstrating a better advantage in stress on the meniscus and cartilage, which can effectively reduce the risk of OA after transplantation.^127^ Zhu *et al*. (2019) suggested that meniscal grafts with porous structures facilitated cell attachment and proliferation and that cartilage had less compressive and shear stress, maintaining the natural morphological characteristics of the meniscus.^128^

The construction of a finite model of the meniscus might be difficult considering the complexity of the tissue structure of the anatomical contact. Some simplification of the contact setup of the meniscus occurred in most studies. Although this obtained data was consistent with clinical results, there is still improvement in the model that requires experimental validation.

#### Ligament Injury

**FIGURE 4:**
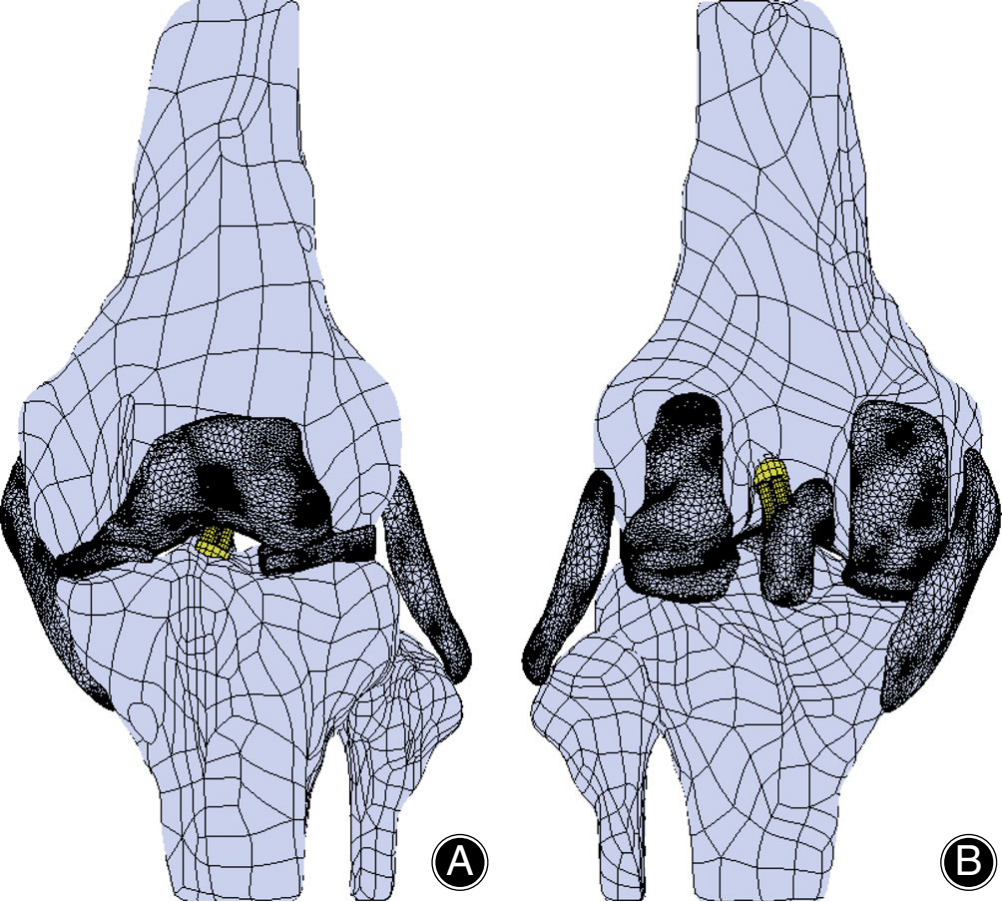
Diagram of the FEM of ACLR in the knee joint. (A) anterior view of the FEM. (B) posterior view of the FEM. ACLR, anterior cruciate ligament reconstruction; FEM, finite element model.

Knee ligament injuries are common in sports, and FEMs help us understand the mechanisms of ligament injuries (Figure 4). Gardiner *et al*. proposed an FEM to predict the strain distribution in the MCL under knee valgus loading, and the maximum strain in the ligament occurred in the posterior region near the joint line, which suggested a higher probability of injury in this region.^11^ Alexander *et al*. found that an incompletely ruptured ACL could significantly affect the load of adjacent ligaments.^129^ PCL rupture could also affect the function of the ACL if left untreated, which showed the importance of early diagnosis and active management of complications after ligament rupture.^130^ A finite element study suggested that the stress increase in the popliteal tendon was greatest after PCL rupture. The tibiofemoral joint contact stress changed significantly during deep squatting, with the medial stress in the patellofemoral joint increasing and the lateral stress decreasing, suggesting that PCL rupture could affect the tibiofemoral joint stress and lead to joint degeneration.^131^ Bernardo *et al*. found a significant increase in the MCL strain after UKA, which might help explain the joint pain after UKA.^132^

Finite element models can also guide surgical reconstruction after a ligament injury. Bartolin *et al*. established an animal simulation model of ACL injury and demonstrated that ACL reconstruction (ACLR) with the internal brace technique could effectively improve the stability of the knee joint.^133^ Different drilling angles of the femoral tunnel affect the bone tunnel and ACL graft stress and strain.^9^ A comparison of techniques for establishing femoral tunnels with ACL reconstruction has been made. The anteromedial technique obtained shorter tunnels with higher graft and tunnel stresses,^134^ and the hybrid transtibial technique had advantages in terms of tunnel length and graft stresses, which might be a better method for establishing femoral tunnels.^135^ A new tunnel protection liner was designed to reduce the stress on the tunnel entrance and grafts, improving the bone–graft interaction at the tunnel entrance, which would avoid long‐term tunnel enlargement and graft wear.^7^ Shu *et al*. demonstrated that a more significant posterior tibial tilt put a greater load on the medial meniscus and increased the risk of anterior ACL reconstruction failure.^136^ The effect of graft stiffness and shape on the tunnel and graft after ACL reconstruction has been investigated, and it was found that high stiffness and oval‐shaped grafts increased the stress on the bone tunnel and thus caused tunnel enlargement and graft wear.^137^ Risvas *et al*. found that increasing the graft pretension and radius could reduce relative knee displacement.^138^ Many finite element studies have also been conducted on medial patellofemoral ligament (MPFL) injuries. MPFL reconstruction alone was sufficient to restore the kinematic function of the knee, and tibial tuberosity displacement was necessary when the distance between the tibial tuberosity and the talocrural groove was too large.^60^ It has been suggested that tibial tuberosity displacement combined with lateral retinaculum release could significantly reduce the contact area and stress in the patellofemoral joint, but there was a risk of lateral patellofemoral instability.^79^ Wierer *et al*. found that the risk of patellar fracture after MPFL reconstruction depended on the location of the patellar tunnel and that invasion of the bone tunnel into the anterolateral patellar cortex increased the risk of patellar fracture.^139^ Vicente *et al*. found that selecting the adductor magnus tendon (AMT) or quadriceps terminations for MPFL reconstruction resulted in lower patellar stress, while anatomical reconstruction increased patellar stress and thus the risk of patellofemoral OA.^140^

Finite element studies related to knee ligament injuries give us a broad idea on sports injury and prevention for future research. In the future, it is crucial to implement finite element simulation in complex sports states. For example, the prevention and protection of ligament injuries in winter sports will be of cross‐generational significance.

### Knee Rehabilitation and Exercises

Finite element analysis can provide more guidelines and suggestions for rehabilitation exercises. FE‐MS models can guide patients’ rehabilitation exercises. Funaro *et al*. analyzed and compared the effects of different rehabilitation activities on the Achilles tendon, suggesting that rehabilitation activities should transition from walking on heels to walking on toes and then to heel dropping with the knee bent, with attention to avoiding jumping on one foot.^61^ Amir *et al*. conducted a finite element analysis of daily and rehabilitation activities in patients with knee OA and found that the medial tibia had greater mechanical stress than the lateral in most daily activities, a result that matched the higher prevalence of OA in the medial compartment.^58^ The rehabilitation exercise of knee extension could effectively strengthen the quadriceps muscle. The stress on the lateral tibial cartilage was greater than that on the medial side, suggesting that this rehabilitation activity could have a promising clinical future.^58^

Knee FEM can also serve as an aid in sports and training, helping athletes to engage in safer training and have better sports performance. Yu *et al*. evaluated the patellofemoral joint loading during the directional lunges of badminton players through MS‐FE modeling, finding that the proper patellofemoral joint loading was more significant when right‐handed players were making a left‐backward lunge.^141^ Esrafilian *et al*. presented a method for rapid musculoskeletal modeling, which was validated with predictive data for studying knee joint dynamics at different levels of mobility in OA patients.^67^ Navacchia *et al*. developed a method to track lower limb joint motion, which in conjunction with an FE‐MS model could effectively estimate the relationship between muscle force and tissue deformation.^77^ This method resolved the conflict between the calculation cost of the MS‐FE model and the model details, suggesting the feasibility of FEMs in muscle force prediction.^77^

Knee finite element modeling in sports training and rehabilitation is being progressively developed. More detailed and complex musculoskeletal FEMs have also enabled better simulation of body functions. The field of post‐knee injury rehabilitation and sports exercise holds great promise for the future.

## Discussion

In this study, a review is presented regarding finite element model construction, model verification, and clinical application of the knee FEM. Material selection and clinical application of the model are highlighted according to different anatomical structures, such as bone, cartilage, meniscus, ligaments, muscles, and tendons, which can provide researchers with methodological guidance for modeling the knee joint.

The number of finite element studies of the knee joint is increasing, and the 3D simulation models are becoming more well developed. Meanwhile, finite element studies of the knee joint have been greatly welcomed. Considering the structural relationships of the knee joint are complex, accurate anatomy and suitable materials are the basis for building an acceptable simulation model. This review provides a reference for the selection of tissue materials for different research purposes and research conditions.

Finite element analysis can effectively simulate tissues’ biomechanical behavior and affect mechanical tests with relatively low cost and risk. However, a larger sample and detailed validation are essential to make the analysis results more reliable. Most finite element studies focus on the pathological state, but the interplay of multiple pathological conditions often accompanies diseases. For example, ACL rupture might be accompanied by meniscal damage, cartilage damage, or avulsion fractures. A simulation model that better matches the actual state of the disease is a challenge to be tackled in the future. Most of the previous studies did not consider the effect of body temperature and joint fluid on the simulation model, and more work is needed in the future to address this issue. In addition, the knee, an important weight‐bearing joint of the lower extremity, can also perform flexion, extension, and rotation activities. There are more complex movement states in advanced competitive sports such as soccer and skiing. The simulation of advanced motion in a more realistic knee model would be a milestone in the field.

## Conclusion and Perspective

In conclusion, this review first summarized various modeling strategies, applications, and characteristics of the knee joint for different research purposes and could serve as a reference for constructing knee joint models and designing research in the future. Finite element modeling of the knee joint provides insight into the biomechanical characteristics of the knee joint, the development of new implant materials, the prediction of knee joint disease, the improvement of surgical techniques, and the guidance on rehabilitation exercises. It is believed that finite element research will bring more surprises and a better outlook for clinical research in the future.

## Conflict of Interest Statement

The authors declare that the research was conducted in the absence of any commercial or financial relationships that could be construed as a potential conflict of interest.

## Author Contributions

Mingyue Yan, Haibo Zhao, and Tengbo Yu contributed to the conception and design of the study. Mingyue Yan wrote the first draft of the manuscript. Ting Liang, Yanchi Bi, and Tianrui Wang looked up and selected literature from the database. Tianrui Wang, Tengbo Yu, and Yingze Zhang supervised the manuscript. All authors contributed to manuscript revision and read and approved the submitted version.

## Funding Information

This work was supported by grants from the National Natural Science Foundation of China (31872310) and Shandong Province Key R&D Program Project (2021SFGC0502).

## Ethical Statement

All authors have contributed to this article. All have reviewed the article and agreed to its publication.
